# Endogenous thrombopoietin promotes non‐small‐cell lung carcinoma cell proliferation and migration by regulating EGFR signalling

**DOI:** 10.1111/jcmm.15314

**Published:** 2020-04-26

**Authors:** Zifang Zou, Xiaoxi Fan, Yang Liu, Yanbin Sun, Xin Zhang, Guanghao Sun, Xuehao Li, Shun Xu

**Affiliations:** ^1^ Department of Thoracic Surgery The First Hospital of China Medical University Shenyang China

**Keywords:** epidermal growth factor, migration, non‐small‐cell lung cancer, PI3K/AKT/mTOR, proliferation, thrombopoietin

## Abstract

Thrombopoietin (TPO) is a haematopoietic cytokine mainly produced by the liver and kidneys, which stimulates the production and maturation of megakaryocytes. In the past decade, numerous studies have investigated the effects of TPO outside the haematopoietic system; however, the role of TPO in the progression of solid cancer, particularly lung cancer, has not been well studied. Exogenous TPO does not affect non‐small‐cell lung cancer (NSCLC) cells as these cells show no or extremely low TPO receptor expression; therefore, in this study, we focused on endogenous TPO produced by NSCLC cells. Immunohistochemical analysis of 150 paired NSCLC and adjacent normal tissues indicated that TPO was highly expressed in NSCLC tissues and correlated with clinicopathological parameters including differentiation, P‐TNM stage, lymph node metastasis and tumour size. Suppressing endogenous TPO by small interfering RNA inhibited the proliferation and migration of NSCLC cells. Moreover, TPO interacted with the EGFR protein and delayed ligand‐induced EGFR degradation, thus enhancing EGFR signalling. Notably, overexpressing TPO in EGF‐stimulated NSCLC cells facilitated cell proliferation and migration, whereas no obvious changes were observed without EGF stimulation. Our results suggest that endogenous TPO promotes tumorigenicity of NSCLC via regulating EGFR signalling and thus could be a therapeutic target for treating NSCLC.

## 1 INTRODUCTION

Lung cancer is the leading cause of cancer‐related deaths worldwide.^1^ There are two main subtypes of lung cancer, small‐cell lung carcinoma and non‐small‐cell lung carcinoma (NSCLC), with NSCLC accounting for approximately 85% of all cases.^2^ Surgery is the most effective treatment strategy for NSCLC; however, many cases are diagnosed at an advanced stage when surgery is no longer possible. Although other treatment options are available, including chemotherapy, radiotherapy, immunotherapy and molecularly targeted therapy,^3^ lung cancer exhibits a very poor prognosis and nearly half of all patients die within one year of diagnosis, with a 5‐year survival rate of 11%.^4^ A better understanding of NSCLC initiation and development mechanisms is urgently needed, and studies are required to identify more oncogenes and suppressor genes as well as targetable gene alterations.

Thrombopoietin (TPO) is a haematopoietic cytokine that is mainly produced by the liver and kidneys and whose main function is to regulate megakaryocyte progenitor expansion and differentiation.^5^ Scientists first successfully purified TPO in 1994; after it was cloned, thrombopoietic agents rapidly entered clinical development. PEG‐rhMGDF and rh‐TPO were the first‐generation thrombopoietic drugs. Soon afterwards, TPO receptor agonists romiplostim and eltrombopag were produced as second‐generation thrombopoietic drugs that had a better efficacy and rare side effects. These agents are mainly used to cure various types of primary or secondary thrombocytopenia, particularly thrombocytopenia caused by chemotherapy. Exogenous TPO acts by binding to the TPO receptor (C‐MPL) and initiating various signal transduction pathways.^6^, ^7^, ^8^ Most solid tumour tissues and cell lines, including NSCLC, do not express C‐MPL or exhibit extremely low expression.^9^, ^10^, ^11^ Consequently, exogenous TPO and TPO receptor agonists cannot affect the progress of these tumours, and thrombopoietic drugs were considered safe to cure thrombocytopenia in most cancer patients. In 1990s, a study reported TPO mRNA expression in some solid tumour cell lines^11^; however, the function of endogenous TPO in solid tumours has generally been neglected. Our study focuses on the endogenous TPO produced by NSCLC cells and whether it affects the occurrence and development of lung cancer.

Epidermal growth factor receptor (EGFR) is a receptor tyrosine kinase belonging to the ErbB family that plays crucial roles in both normal physiological and pathological processes.^12^ EGFR is activated upon binding to its ligands, after which a series of conformational changes occur in both the extracellular and intracellular domains.^13^ This leads to the *trans*‐autophosphorylation of tyrosine residues in the C‐terminal regulatory domain and initiates intracellular signalling cascades,^13^ including numerous signalling pathways such as the PI3K/AKT, RAS/MAPK, STAT and JNK pathways.^14^ This process can regulate cell apoptosis, proliferation, migration and differentiation, which have important effects on cancer phenotypes.^15^ Increased EGFR expression has been found in multiple types of human cancers, including lung, breast, colon, oral and kidney cancers.^16^ Thus, EGFR serves as a prognostic indicator in many types of cancer as its high expression is related to poor prognosis. EGFR mutations in lung cancer were first detected in 2004 and have subsequently been widely examined, resulting in the development of new therapeutic strategies for patients with NSCLC. EGFR tyrosine kinase inhibitors and monoclonal antibodies against EGFR have become critical for treating NSCLC as they prolong the survival of patients with advanced NSCLC.^17^ However, EGFR‐targeted therapy still has various limitations in clinical practice that necessitate further studies of EGFR and EGFR signalling.

Co‐expression of EGFR and its ligands is commonly found in primary lung cancer.^18^ EGF is the best characterized EGFR‐specific ligand serving as agonist of EGFR signalling.^19^ Its binding to the extracellular domain of EGFR initiates EGFR signalling which, once activated, is controlled by a negative feedback mechanism to avoid constant activation.^20^, ^21^ Endocytic trafficking, which involves internalization, endosomal sorting and lysosomal degradation, can attenuate EGFR signalling by removing and down‐regulating activated receptors from the plasma membrane^22^ and plays an important role in regulating the extent and duration of EGFR signalling.^23^, ^24^ Endocytic trafficking and degradation is a hot topic in EGFR signalling research because of its crucial role and high degree of complexity. An increasing number of studies have attempted to determine the behaviour of this complex process; however, the exact mechanism remains unclear.

In this study, we investigated the expression of TPO in NSCLC tissues and its clinicopathological relevance, as well as explored the biological role of endogenous TPO in NSCLC cells. We found that TPO interacts with EGFR and influences EGFR signalling. This haematopoietic cytokine may enhance the development and progression of NSCLC and may be a useful therapeutic target.

## 2 MATERIALS AND METHODS

### 2.1 Patients and specimens

A total of 150 paired NSCLC/normal specimens were obtained from patients with NSCLC who underwent surgical resection at the Department of Thoracic Surgery of the First Hospital of China Medical University between 2014 and 2016. No patients underwent chemotherapy or radiotherapy prior to surgery. The normal liver and kidney specimens were obtained from the Pathology Archive of the First Affiliated Hospital of China Medical University. Written informed consent was provided by each patient, and the study was approved by the Medical Research Ethics Committee of China Medical University.

### 2.2 Immunohistochemistry and immunofluorescence

Assays were performed as described previously.^25^ For immunohistochemistry, all specimen sections were incubated with anti‐TPO rabbit monoclonal antibodies (1:100; ab196026; Abcam) overnight at 4°C. Staining intensity represented TPO expression and was scored as follows: 0 (no staining), 1 (weak), 2 (moderate) or 3 (high). Percentage scores were assigned as follows: 1 (1%‐25%), 2 (26%‐50%), 3 (51%‐75%) and 4 (76%‐100%). The two scores were multiplied for each specimen to give a final score of 0‐12. Specimens with scores >6 were considered TPO‐positive, whilst those with scores ≤ 6 were considered TPO‐negative. For immunofluorescence, cells were incubated with anti‐TPO rabbit monoclonal antibodies (1:50; ab196026; Abcam) or anti‐EGFR rabbit monoclonal antibodies (1:50; #4267; Cell Signaling Technology) overnight at 4°C. Images were acquired with an Olympus FV3000 confocal laser scanning microscope (Tokyo).

### 2.3 Cell lines and cell culture

A549, H1299, H460, SK‐MES‐1 and H292 cells were purchased from the Cell Bank of the China Academy of Sciences (Shanghai). HBE cells were obtained from the American Type Culture Collection (ATCC). All cells were cultured according to the instructions of the ATCC/CTCC. All media were purchased from Gibco, and FBS was purchased from Clark.

### 2.4 Plasmid transfection and small interfering RNA treatment

Transfection was performed with Lipofectamine 3000 reagent (Invitrogen) according to the manufacturer's instructions. For TPO overexpression, pCMV6‐Myc‐DDK (#PS100001) and pCMV6‐Myc‐DDK‐thrombopoietin (#RC221324) were purchased from OriGene. For TPO knockdown, TPO‐specific small interfering RNA (siRNA) and negative control siRNA were purchased from RiboBio. The TPO‐siRNA sequence was GGATACACGAACTCTTGAA.

### 2.5 Western blot

Total protein was extracted using lysis buffer (Beyotime Biosciences) containing PMSF (Beyotime Biosciences) and phosphatase inhibitor cocktail (Biotool, Shanghai, China) and then quantified according to the Bradford method. Total protein samples (60 μg) were separated by 10% sodium dodecyl sulphate‐polyacrylamide gel electrophoresis, transferred to polyvinylidene fluoride membranes (Millipore) and incubated in TBST with 5% skim milk (BD Biosciences) at 37°C for 2 h and then different primary antibodies overnight at 4°C. The membranes were washed three times with TBST for 10 min each time and incubated with peroxidase‐conjugated antimouse or anti‐rabbit IgG (1:2000; ZSGB‐BIO) at 37°C for 2 h. Protein bands were visualized with a bio‐imaging system (DNR), and relative protein expression was determined using GAPDH or beta‐actin as a loading control. Thrombopoietin (ab196026) antibodies were purchased from Abcam. Cyclin E1 (#4129), cyclin E2 (#4132), RhoA (#2117), RhoC (#3430), MYC (#2278), EGFR (#4267), P‐EGFR (Tyr1068; #3777), P‐mTOR (Ser2448; #5536), mTOR (#2983), P‐AKT (Ser473; #4060) and AKT (#4865) antibodies were purchased from Cell Signaling Technology. CDK2 (10122‐1‐AP), P27 (25614‐1‐AP), C‐Myc (10828‐1‐AP), beta‐actin (60008‐1‐ Ig) and GAPDH (60004‐1‐Ig) antibodies were purchased from Proteintech. All experiments were repeated independently at least three times.

### 2.6 RNA extraction and real‐time RT‐PCR

These assays were performed as described previously^26^ with the following primer sequences: *EGFR*, forward 5′‐GGAGAACTGCCAGAAACTGACC‐3′ and reverse 5′‐GCCTACAGCACACTGGTTG‐3′; *TPO*, forward 5′‐AACTGCAAGGCTAACGCTGT‐3′ and reverse 5′‐GACATGGGAGTCACGAAGCA‐3′; and *GAPDH*, forward 5′‐GGAGCGAGATCCCTCCAAAAT‐3′ and reverse 5′‐GGCTGTTGTCATACTTCTCATGG‐3′. All experiments were repeated independently at least three times.

### 2.7 Cell proliferation assay and colony formation assay

Assays were performed as described previously.^27^ For the Cell Counting Kit‐8 (CCK‐8) assay, 3000 cells were added to each well of a 96‐well plate containing 100 μL medium. Absorbance was quantified at 450 nm every 24 h for 5 days to generate a growth curve. For the colony formation assay, 1000 cells were added to each 60‐mm cell culture dish containing 4 mL medium and incubated in a 5% CO_2_ incubator at 37°C for 10‐14 days. Images were acquired with a bio‐imaging system (DNR). All experiments were repeated independently at least three times.

### 2.8 Cell migration analysis

Assays were performed as described previously.^25^ Cells were collected by trypsin digestion 24 h after transfection. For A549 or H1299 cells, 80 000 or 60 000 cells were placed into the upper chamber in 200 μL 1640 medium containing 2% FBS, and 800 μL 1640 medium containing 10% FBS was placed in the lower chamber. Cells were stained after incubation for 20 h. All experiments were repeated independently at least three times.

### 2.9 Evaluation of TPO in cell medium by ELISA

The cell lines were cultured in RPMI 1640 medium containing 10% FBS. After 48 h, the medium of each cell line was collected for TPO detection. TPO was evaluated using an ELISA kit (KeyGen Biotech, Nanjing, China) according to the manufacturer's instructions. The minimum detection range was 62 pg/mL. All experiments were repeated independently at least three times.

### 2.10 Mass spectrometric analysis

A549 cells were collected 48 h after transfection with pCMV6‐Myc‐DDK‐TPO plasmids. Myc‐tagged fusion proteins were captured by incubation with anti‐Myc antibodies (# 2276; Cell Signaling Technology) and ProteinA + G Agarose beads (P2012; Beyotime Biosciences) overnight at 4°C. The negative control groups were incubated with anti‐IgG mouse antibodies (A7028; Beyotime Biosciences) and ProteinA + G Agarose beads. After dissociation from the beads, the denatured immunocomplexes were run on an 10% SDS‐PAGE gel. The selected bands were sent to Shanghai Applied Protein Technology for mass spectrometric analysis after Coomassie blue staining.

### 2.11 Co‐immunoprecipitation assay

Assays were performed as described previously.^28^ To verify interaction between transfected TPO and EGFR, A549 and H1299 cells were collected 48 h after transfection with pCMV6‐Myc‐DDK‐TPO plasmids, and then, cell lysates were immunoprecipitated with anti‐Myc antibodies (# 2276; Cell Signaling Technology) or control IgG mouse antibodies. To verify interaction between endogenous TPO and EGFR, cell lysates were immunoprecipitated with anti‐TPO antibodies (sc‐374045; Santa Cruz Biotechnology) or control IgG mouse antibodies. All experiments were repeated independently at least three times.

### 2.12 Growth factors

Recombinant human EGF and recombinant human TPO were purchased from PeproTech (AF‐100‐15‐100) and R&D Systems (288‐TP‐005/CF), respectively.

### 2.13 Statistical analysis

SPSS 17.0 software (SPSS, Inc) and GraphPad Prism 6.0 software were used for statistical analyses. The chi‐squared test was used to test the association between TPO expression and clinicopathological parameters. Between‐group differences were analysed using paired Student's *t* tests. *P* values of <0.05 were considered to represent a significant difference.

## 3 RESULTS

### 3.1 TPO is highly expressed in NSCLC tissues and has significant clinical relevance

**Figure 1:**
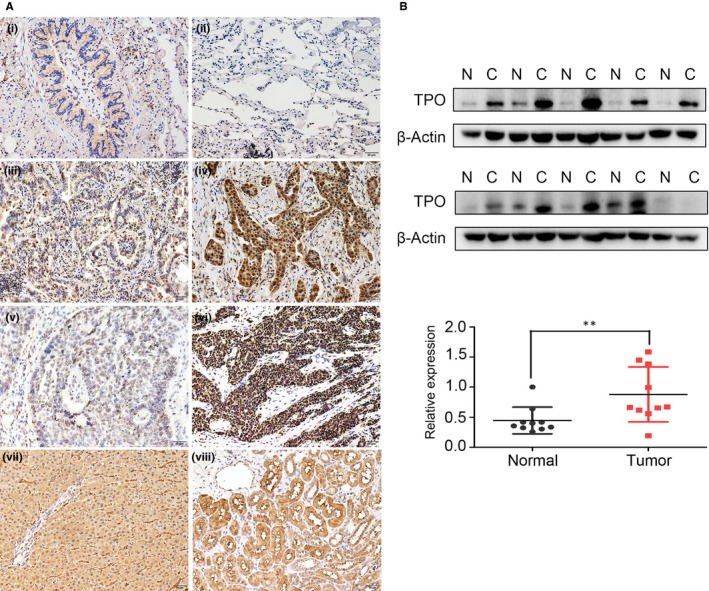
TPO is highly expressed in NSCLC tissues A, TPO expression was negative in (a) paired normal bronchial and (b) alveolar epithelial cells but was positive in NSCLC tissues: (c) highly differentiated adenocarcinoma; (d) poorly differentiated adenocarcinoma; (e) highly differentiated squamous carcinoma; and (f) poorly differentiated squamous carcinoma; (g) normal liver tissue; and (h) normal kidney tissue. Magnification, ×200. B, Western blot analysis indicated that TPO was highly expressed in fresh non‐small‐cell lung cancerous tissues (C) compared to corresponding non‐cancerous tissues (N). Relative quantification of protein expression was analysed by ImageJ software. *P < 0.05; **P < 0.01

We performed immunohistochemical analyses on 150 paired NSCLC/normal tissues, including 66 squamous cell carcinoma and 84 adenocarcinoma samples. TPO was highly expressed in NSCLC tissues compared to peritumour tissues and localized in both the cytoplasm and nuclei (Figure 1A). Of the 66 squamous cell carcinoma samples, 41 were TPO‐positive, whereas 50 of the 84 adenocarcinoma samples were TPO‐positive. As shown in Table 1, TPO expression was also positively correlated with clinicopathological parameters of NSCLC patients, including differentiation (*P* = 0.015), P‐TNM stage (*P* < 0.01), lymph node metastasis (*P* < 0.01) and tumour size (*P* < 0.01). We also stained 6 tissue samples of normal liver and kidney with the same antibody as positive controls (Figure 1A). Furthermore, we detected TPO expression in 10 paired fresh NSCLC and corresponding non‐cancerous tissues by Western blotting, finding that TPO was highly expressed in NSCLC specimens compared to the surrounding normal tissue (Figure 1B).

**Table 1:** Correlation of TPO expression with clinicopathological parameters of NSCLC patients

Clinicopathological characteristics	Total N	TPO‐negative		TPO‐positive		*P*‐value
Age (years)						
≤60	68	26		42		0.802
>60	82	33		49		
Gender						
Male	99	36		63		0.3
Female	51	23		28		
Histological type						
Squamous cell carcinoma	66	25	41			0.747
Adenocarcinoma	84	34	50			
Differentiation						
Well‐Moderate	86	41		45		0.015
Poor	64	18		46		
Tumour size (cm)						
≤3	56	35		21		<0.01
>3	95	24		71		
Lymph node metastasis						
Negative	90	46		44		<0.01
Positive	60	13		47		
TNM stage						
I‐IIA	75	44		31		<0.01
IIB‐III	75	15		60		

### 3.2 TPO expression and subcellular localization in NSCLC cell lines

**Figure 2:**
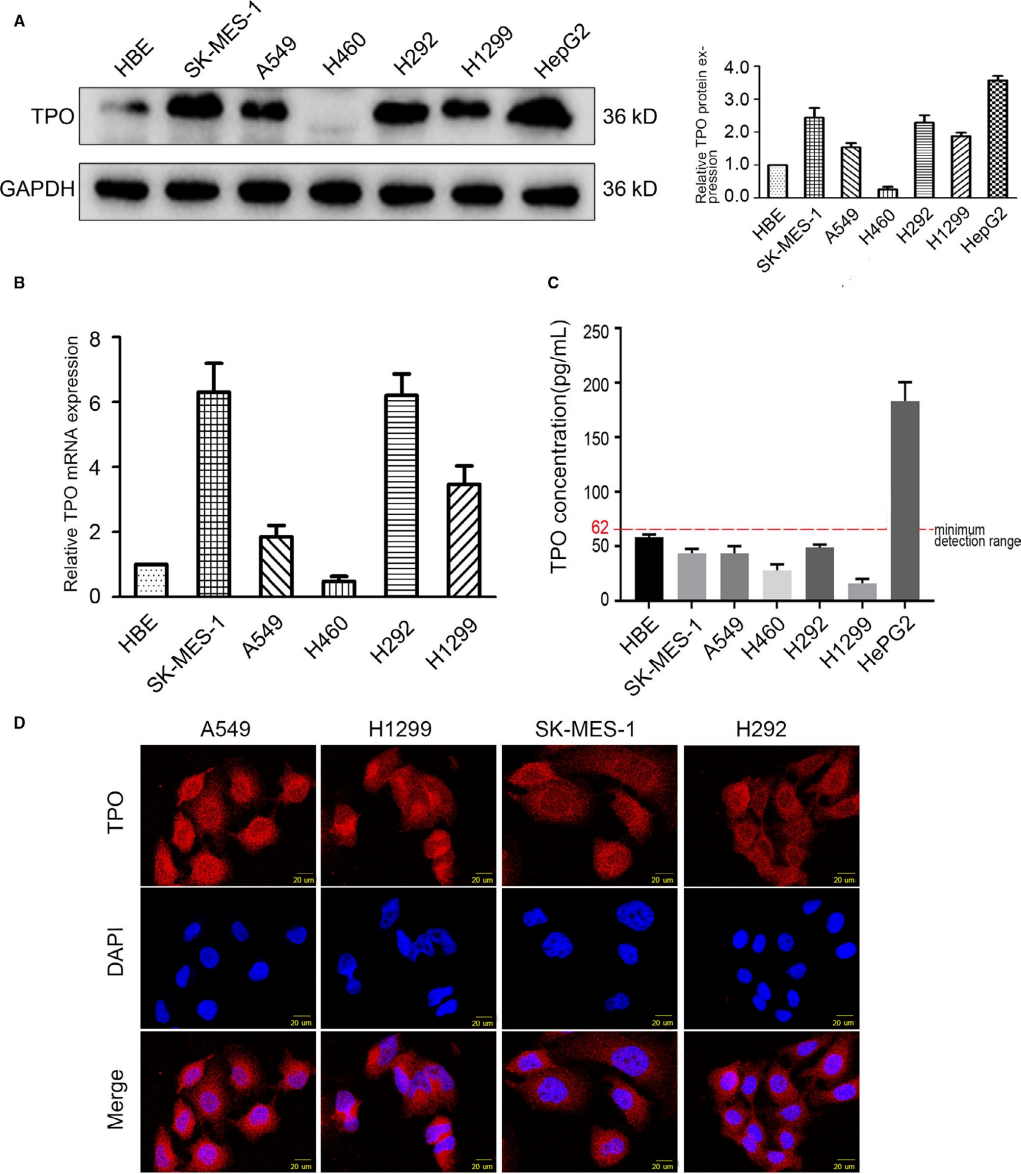
TPO expression and subcellular localization in NSCLC cell lines. A, Western blot analysis indicated that TPO protein level was increased in A549, H1299, SK‐MES‐1 and H292 cells compared to HBE cells (normal bronchial epithelial cells) but was weak in H460 cells. HepG2 cells were used as the positive control. Relative quantification of protein expression was analysed by ImageJ software. The value of the control group HBE was set to ‘1’. B, RT‐PCR analysis indicated that TPO mRNA level was increased in A549, H1299, SK‐MES‐1 and H292 cells compared to HBE cells (normal bronchial epithelial cells) but was weak in H460 cells. The value of the control group HBE was set to ‘1’.C, ELISA results depict that TPO concentration in the media of HBE, A549, H1299, H460, SK‐MES‐1 and H292 cells was below the minimum detection range. HepG2 cells were used as the positive control. D, Immunofluorescence assays demonstrated that TPO was localized in both the cytoplasm and nucleus of the NSCLC cell lines, mainly in the cytoplasm and partially in the nucleus. Magnification, ×400. Data are presented as the mean ± SD of three independent experiments

TPO protein and mRNA expression in 5 NSCLC cell lines and normal bronchial epithelial HBE cells was examined, showing that TPO expression was increased in A549, H1299, SK‐MES‐1 and H292 cells compared to that in HBE cells but was weakly expressed in H460 cells (Figure 2A,B). We also detected whether the secreted TPO exists in the medium of these NSCLC cell lines and HBE cells. ELISA results revealed that there was no detectable TPO secreted from NSCLC or HBE cells (Figure 2C). Immunofluorescence analysis of A549, H1299, SK‐MES‐1 and H292 cells showed that TPO was localized in both the cytoplasm and nucleus (Figure 2D). As above, we found that TPO is highly expressed in most NSCLC cell lines compared to HBE cells at both the mRNA and protein levels but not secreted to the medium. NSCLC tissue and cell lines have been previously proven to have extremely low or almost negligible TPO receptor (C‐MPL) expression, and NSCLC cells are not affected by exogenous TPO.^9^, ^10^, ^11^ Hence, our research group focused on the endogenous TPO produced by NSCLC cells. A549 and H1299 cells were chosen for the subsequent experiments because of their moderate TPO expression. Human hepatocellular carcinoma HepG2 cells were used as the positive control in Western blot, ELISA and immunofluorescence assays (Figure S1A) as it is well known that HepG2 cells produce and secrete TPO.^29^

### 3.3 TPO suppression inhibits NSCLC cell proliferation and migration

**Figure 3:**
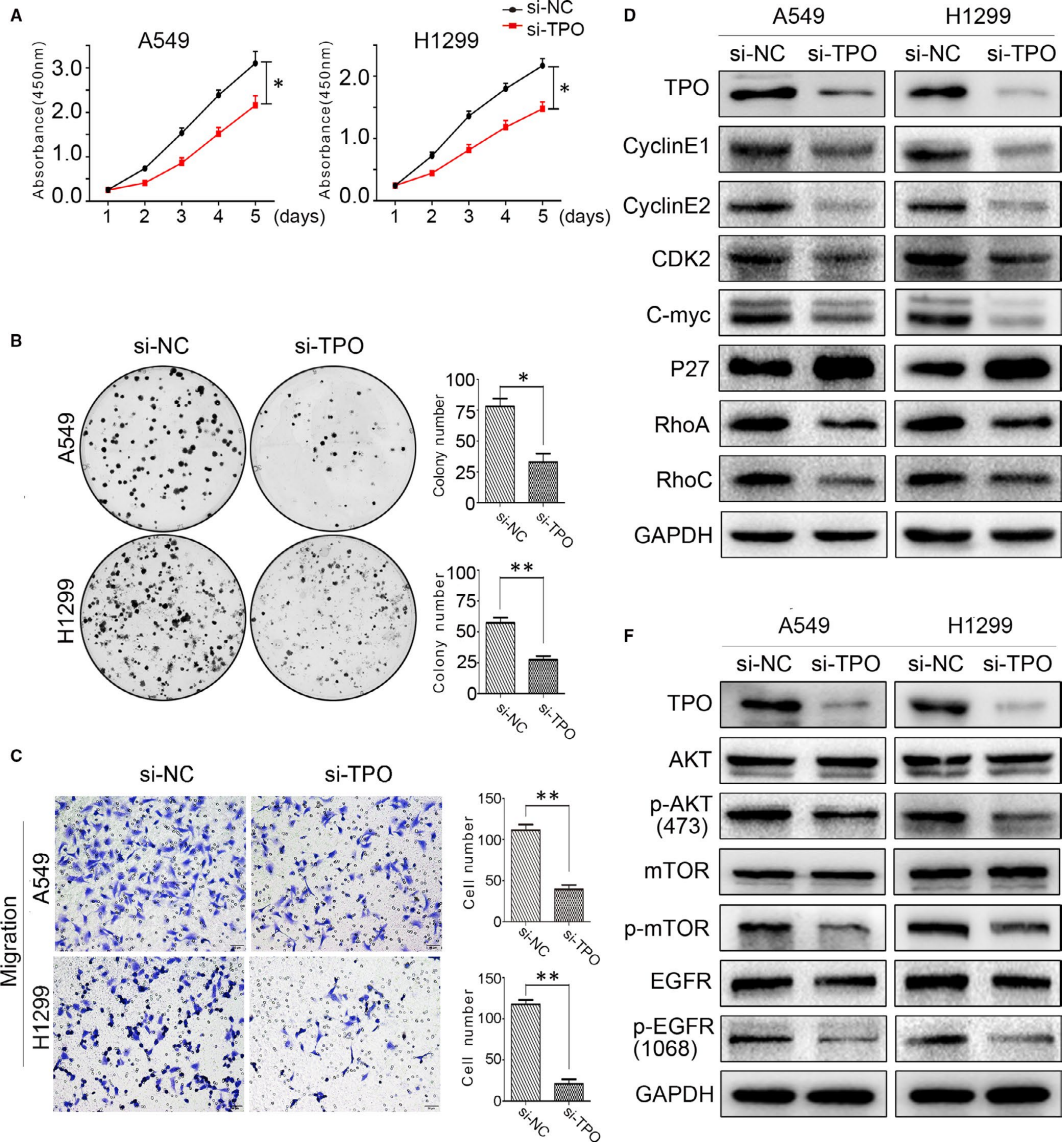
TPO suppression inhibits NSCLC cell proliferation and migration. A, B, CCK‐8 and colony formation assays demonstrated that A549 and H1299 cell proliferation was down‐regulated when TPO was suppressed. C, Transwell assays showed that TPO knockdown inhibited A549 and H1299 cell migration. Magnification, ×200. D, Cyclin E1, cyclin E2, CDK2, c‐Myc, P27, RhoA and RhoC protein levels were detected by Western blotting when TPO was down‐regulated in A549 and H1299 cells. Relative quantification of protein expression was analysed by ImageJ software (Figure S2). E, AKT, P‐AKT (Ser473), mTOR, P‐mTOR (Ser2448), EGFR and P‐EGFR (Tyr1068) protein levels were detected by Western blotting when TPO was down‐regulated in A549 and H1299 cells. Relative quantification of protein expression was analysed by ImageJ software (Figure S2). *P < 0.05; **P < 0.01. Data are presented as the mean ± SD of three independent experiments

As TPO expression was correlated with NSCLC progression, we analysed whether TPO affects the biological functions of NSCLC cells. We used short interfering RNA (siRNA) to suppress TPO expression in A549 and H1299 cells and used Cell Counting Kit‐8 (CCK‐8) assay and colony formation assay to examine the growth and colony formation abilities of these cells. The results showed that down‐regulating TPO suppressed the proliferation of these two cell lines (Figure 3A and B). We also detected the protein levels of molecules involved in proliferation; cyclin E1, cyclin E2, CDK2 and c‐Myc were down‐regulated and P27 was up‐regulated when TPO was suppressed (Figure 3D). Transwell migration assays showed that suppressing TPO inhibited the migration of A549 and H1299 cells compared to control cells (Figure 3C). Consistently, the expression of the migration‐related proteins RhoA and RhoC was down‐regulated when TPO was knocked down (Figure 3D). As we found that normal bronchial epithelial HBE cells express detectable levels of TPO, we also suppressed TPO expression in HBE cells by siRNA; however, CCK‐8, colony formation and Transwell assays showed no significant differences. These results suggest that the effect of TPO on proliferation and migration is not generalized but cancer‐specific (Figure S1D–F).

### 3.4 TPO overexpression does not affect the biological functions of NSCLC cells

We predicted that TPO overexpression may promote the biological functions of NSCLC cells; however, no positive results were obtained initially. The results of the CCK‐8, colony formation and Transwell assays for A549 and H1299 cells transfected with the TPO plasmid showed no significant differences compared to the control groups (data not shown).

### 3.5 TPO interacts with EGFR and influences EGFR/ PI3K/AKT/mTOR signalling

**Figure 4:**
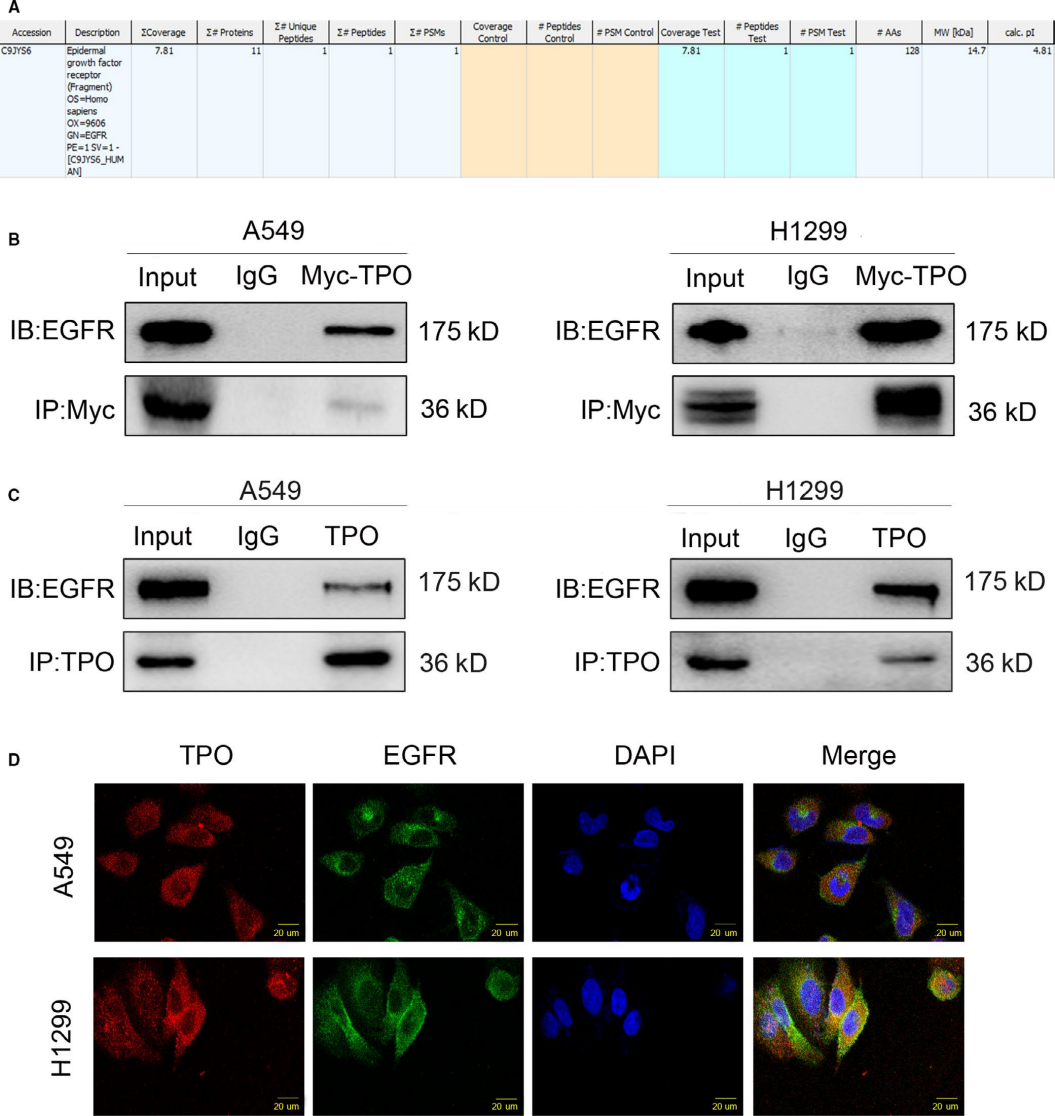
TPO interacts with the EGFR protein. A, Mass spectrometric analysis predicted that TPO could interact with EGFR. B, Interaction between transfected TPO and EGFR was verified by co‐immunoprecipitation assays in A549 and H1299 cells. A549 and H1299 cells were collected 48 h after transfection with pCMV6‐Myc‐DDK‐TPO plasmids. Cell lysates were immunoprecipitated with anti‐Myc antibodies (# 2276; Cell Signaling Technology) or control IgG and examined by anti‐EGFR antibody (#4267; Cell Signaling Technology) and anti‐Myc antibodies (# 2278; Cell Signaling Technology). C, Interaction between endogenous TPO and EGFR was verified by co‐immunoprecipitation assays in A549 and H1299 cells. Cell lysates were immunoprecipitated with anti‐TPO antibody (sc‐374045; Santa Cruz Biotechnology) or control IgG and examined by anti‐EGFR antibody (#4267; Cell Signaling Technology) and anti‐TPO antibody (ab196026; Abcam). D, Immunofluorescence staining in A549 and H1299 cells showed that endogenous TPO and EGFR were colocalized in the cytoplasm. Magnification, ×400

We next evaluated the changes in some critical signalling pathways related to cell proliferation and migration and found that P‐AKT (Ser473) and P‐mTOR (Ser2448) protein levels were significantly down‐regulated when TPO was suppressed (Figure 3E). Meanwhile, no visible changes were observed in P‐ERK, P‐MEK, P‐P65, P‐JNK or active β‐catenin levels. Mass spectrometric analysis enabled us to identify EGFR as one among the many TPO‐interacting proteins (Figure 4A). As EGFR is a key regulator of PI3K/AKT/mTOR signalling,^30^ we verified the interaction between transfected and endogenous TPO and EGFR in A549 and H1299 cells by co‐immunoprecipitation assays (Figure 4B,C) and detected the change in P‐EGFR (Tyr1068) and EGFR levels when TPO was suppressed (Figure 3E). Interestingly, the P‐EGFR level changed significantly with TPO expression but the EGFR level showed no notable change. Immunofluorescence analysis also showed TPO and EGFR to be colocalized in the cytoplasm (Figure 4D). These data suggest that TPO affects NSCLC cells by regulating EGFR/ PI3K/AKT/mTOR signalling.

### 3.6 TPO influences EGFR signalling by delaying ligand‐induced EGFR degradation

**Figure 5:**
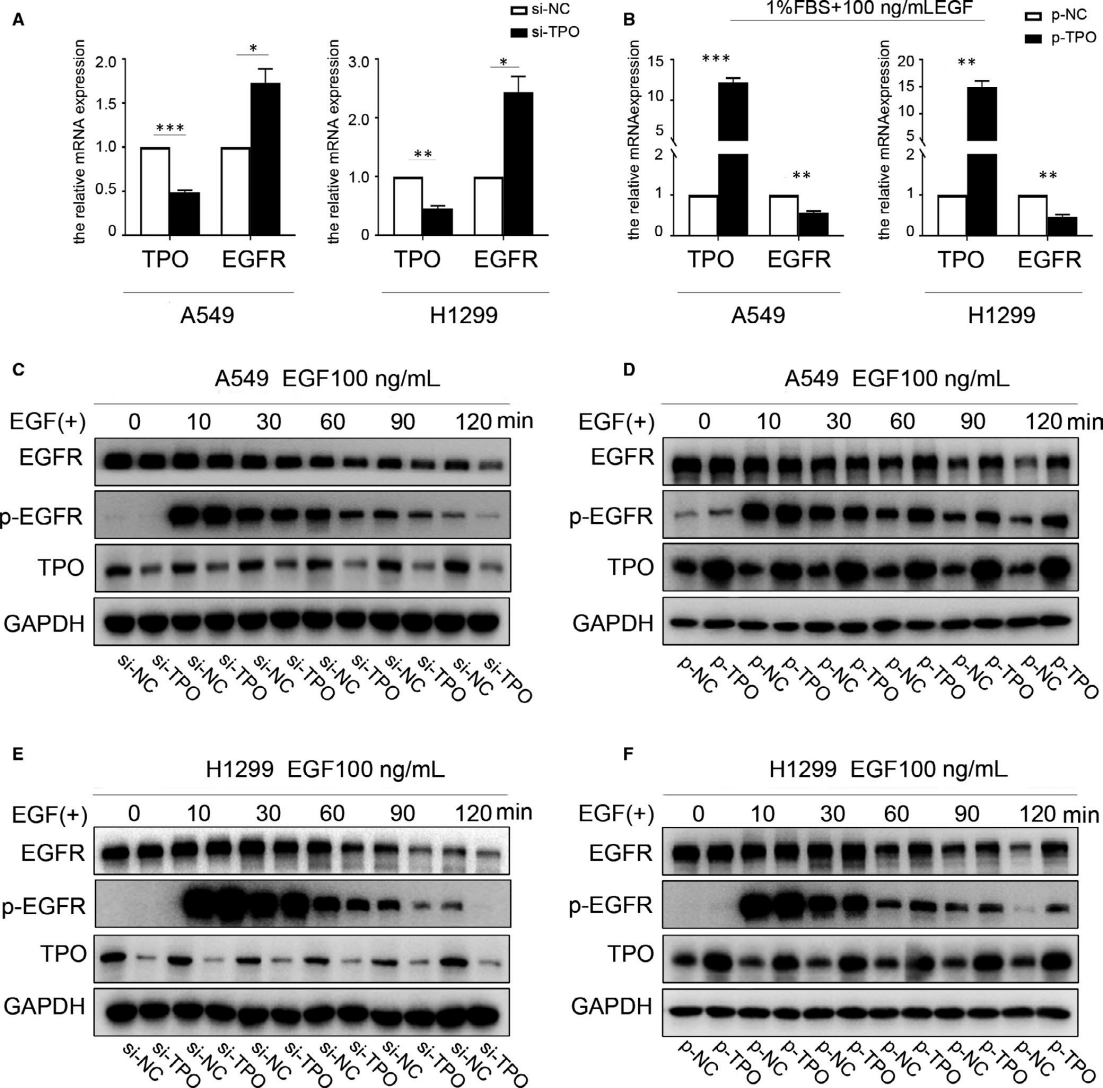
TPO influences EGFR signalling by delaying ligand‐induced EGFR down‐regulation. A, EGFR mRNA expression in case of TPO down‐regulation in A549 and H1299 cells was detected by RT‐PCR. B, EGFR mRNA expression in case of TPO overexpression in EGF‐stimulated A549 and H1299 cells was detected by RT‐PCR. A549 and H1299 cells were serum‐starved overnight and pre‐treated with 150 ng/mL cycloheximide for 1 h before EGF stimulation. We used 100 ng/mL of EGF to activate EGFR signalling and detected EGFR and p‐EGFR (Tyr1068) protein levels at the indicated time‐points. C, E, Suppressing TPO in A549 and H1299 cells accelerated the down‐regulation of EGFR and EGFR signalling after EGF stimulation. D, F, Overexpressing TPO in A549 and H1299 cells attenuated the down‐regulation of EGFR and EGFR signalling and led to sustained EGFR signalling activation in response to EGF stimulation. *P < 0.05; **P < 0.01; ***P < 0.001. Data are presented as the mean ± SD of three independent experiments

To investigate the mechanism via which TPO regulates EGFR signalling, we first assessed EGFR mRNA levels by RT‐PCR when TPO was knocked down. However, in contrast, EGFR mRNA level was up‐regulated compared to that in the control group (Figure 5A). These results indicated that TPO influences EGFR signalling at the post‐translational level. We then explored the effect of TPO on ligand‐induced EGFR activation and degradation. A549 and H1299 cells were serum‐starved overnight and then stimulated with 100 ng/mL EGF for different lengths of time (0, 10, 30, 60, 90 and 120 min), with TPO overexpressed or knocked down at each time‐point. Cycloheximide (150 ng/mL) was used to block protein synthesis before EGF stimulation. EGFR and P‐EGFR (Tyr1068) levels were detected by Western blotting at the indicated time‐points, showing that EGFR signalling was nearly fully activated 10 min after EGF stimulation and that EGFR and P‐EGFR (Tyr1068) protein levels continued to decrease from 30 to 120 min. When new protein synthesis was inhibited, the change in EGFR and P‐EGFR protein levels between 30 and 120 min represented the degradation rate of the receptor and its signalling. We found that EGF‐induced EGFR and P‐EGFR degradation was delayed and impaired in TPO‐overexpressed cells (Figure 5D,F), whereas the opposite effects were observed when TPO was suppressed (Figure 5C,E). Based on the above‐mentioned results, we thought that the transcription change of EGFR is a compensation response due to the change in the EGFR degradation rate. Our study also demonstrated that TPO overexpression becomes functional in EGF‐stimulated NSCLC cells, which is discussed in detail below. Hence, we also analysed the EGFR mRNA level in EGF‐stimulated A549 and H1299 cells, and found it to be down‐regulated when TPO was overexpressed (Figure 5B). Thus, our findings indicate that TPO is a new regulator of EGFR endocytic trafficking and degradation, which enhances EGFR stability and increases the duration and intensity of EGFR signalling.

### 3.7 TPO overexpression facilitates the proliferation and migration of EGF‐stimulated NSCLC cells

**Figure 6:**
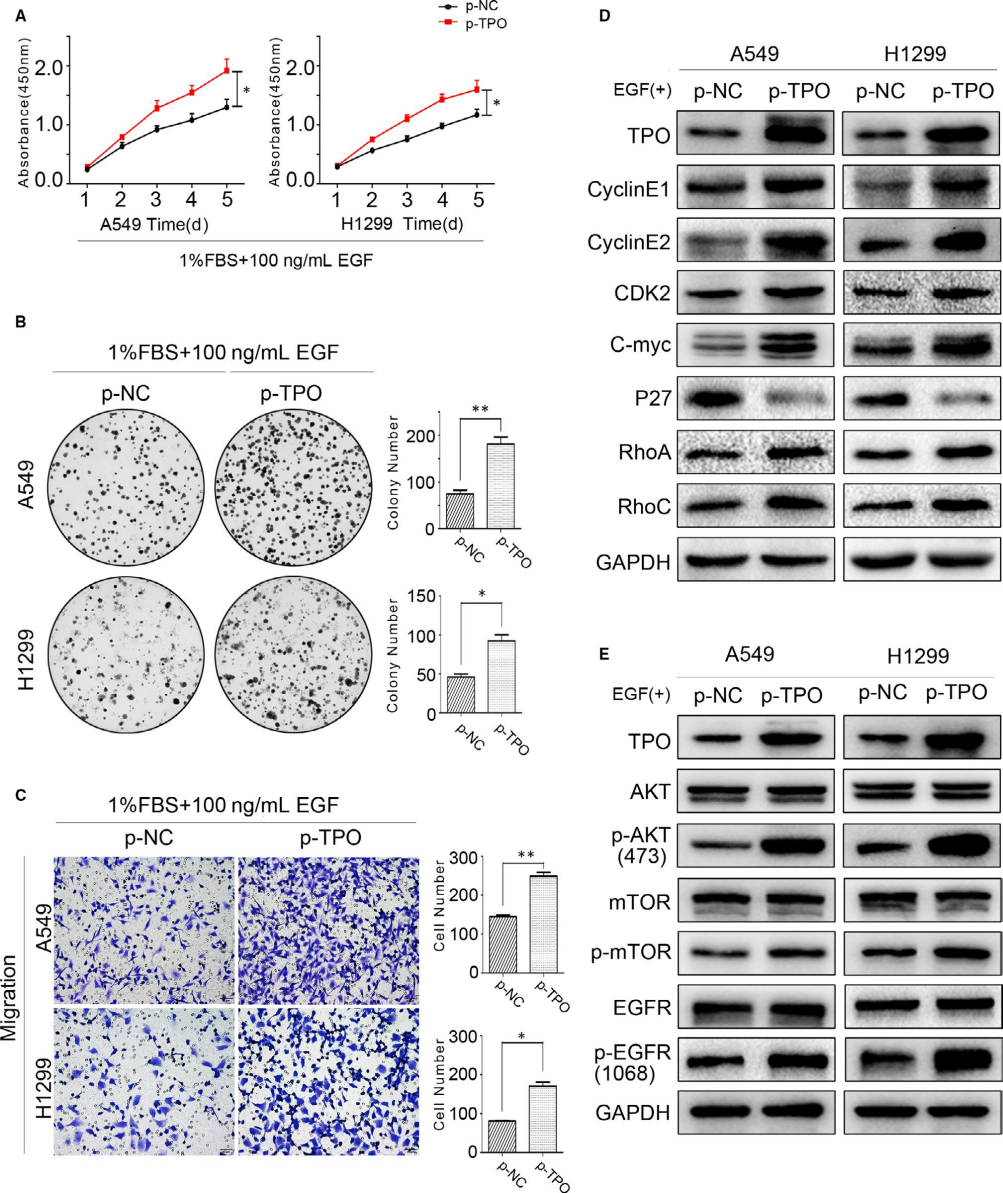
TPO overexpression facilitates the proliferation and migration of EGF‐stimulated NSCLC cells. A549 and H1299 cells were cultured in 1% FBS and 100 ng/mL EGF after transfection with TPO plasmids. A, B, TPO overexpression enhanced A549 and H1299 cell proliferation and colony formation under EGF stimulation, as detected by CCK‐8 and colony formation assays. C, Transwell assays showed that TPO overexpression facilitated the migration of A549 and H1299 cells under EGF stimulation. Magnification, ×200. D, Cyclin E1, cyclin E2, CDK2, c‐Myc, P27, RhoA and RhoC protein levels were detected by Western blotting when TPO was overexpressed in EGF‐stimulated A549 and H1299 cells. Relative quantification of protein expression was analysed by ImageJ software (Figure S2). E, AKT, P‐AKT (Ser473), mTOR, P‐mTOR (Ser2448), EGFR and P‐EGFR (Tyr1068) protein levels were detected by Western blotting when TPO was overexpressed in EGF‐stimulated A549 and H1299 cells. Relative quantification of protein expression was analysed by ImageJ software (Figure S2). *P < 0.05; **P < 0.01. Data are presented as the mean ± SD of three independent experiments

When A549 and H1299 cells were transfected with TPO plasmids, no obvious changes in biological function were observed. The results shown above suggested that TPO affects the biological functions of NSCLC cells by increasing EGFR stability, delaying its down‐regulation and enhancing its signalling. As A549 and H1299 cells contain wild‐type EGFR, TPO overexpression may not promote their proliferation and migration as EGFR signalling and endocytic trafficking activity are relatively low. Thus, we stimulated A549 and H1299 cells with 100 ng/mL EGF, with TPO overexpression enhancing the proliferation and migration of these NSCLC cells (Figure 6A–C). We also detected proliferation‐, migration‐ and EGFR/PI3K/AKT/mTOR‐related proteins by Western blotting, finding that alterations in cyclin E1, cyclin E2, CDK2, c‐Myc, RhoA, RhoC, P‐EGFR (Tyr1068), P‐AKT (Ser473) and P‐mTOR (Ser2448) protein levels were consistent with TPO expression in EGF‐stimulated NSCLC cells, whereas the opposite effect was observed on P27 levels, as expected (Figure 6D,E). These data further indicate that TPO influences the biological function of NSCLC cells by regulating EGFR signalling.

## 4 DISCUSSION

As a haematopoietic cytokine, TPO is mainly expressed in hepatic parenchymal cells and renal tubular cells. The lack of TPO receptor (C‐MPL) is implicated in most solid tumour cells including NSCLC cells, and hence, these are not affected by exogenous TPO that acts by binding to this receptor.^9^, ^10^, ^11^ We verified this conclusion using exogenous rh‐TPO to stimulate A549 and H1299 cells. CCK‐8 and Transwell results showed that rh‐TPO does not affect the proliferation or migration of these two cells (Figure S1B,C). Our study indicated that TPO is highly expressed in NSCLC tissues and cell lines but does not act like a typical cytokine. During our research, we found that TPO is well expressed but is not secreted in many NSCLC cells, which is different from the TPO expression in hepatic or renal cells. This is an interesting phenomenon, and thus, we focused on this endogenous TPO in NSCLC cells. High expression of endogenous TPO was positively correlated with differentiation, P‐TNM stage, lymph node metastasis and tumour size. Furthermore, we investigated the biological functions of endogenous TPO in NSCLC cells. TPO suppression was found to inhibit the proliferation and migration of NSCLC cells by regulating cyclin E1, cyclin E2, CDK2, P27, RhoA, RhoC and c‐Myc protein levels. However, TPO overexpression initially had no effect on these cells.

Our results identified endogenous TPO as a novel regulator of EGFR degradation and EGFR/ PI3K/AKT/mTOR signalling. When EGFR is activated upon binding to its ligands, there are two destinations for EGFR‐ligand complexes via endocytic trafficking: (a) sorting to the lysosome, which terminates signalling; or (b) recycling back to the plasma membrane, which allows the signalling pathway to be constantly activated. Previous studies have shown that NSCLC cells synthesize and secrete EGF, creating an autocrine loop ^31^, ^32^; however, without exogenous EGF stimulation, EGFR signalling activity is relatively low in NSCLC cells containing wild‐type EGFR. In this condition, endocytic trafficking prolongs signalling by reducing the EGFR degradation rate, and activated EGFR mainly undergoes internalization via the clathrin‐mediated endocytosis (CME) route, which tends to recycle EGFR back to the plasma membrane. Consistent with our observations, TPO interacts with EGFR and impairs its degradation, thus enhancing EGFR signalling and NSCLC development. We thought that EGFR and its signalling were saturated with TPO in the absence of exogenous EGF stimulation, and hence, there was hardly any opportunity for overexpressed TPO to further delay EGFR endocytic trafficking and down‐regulation when its degradation rate was already low. Therefore, we stimulated A549 and H1299 cells with exogenous EGF to up‐regulate EGFR signalling, after which the overexpressed TPO became functional. These results further demonstrate that TPO affects the biological function of NSCLC cells by regulating EGFR degradation and its signalling.

In view of our current findings, the influence of TPO on EGFR signalling activity could be attributed to the regulation of EGFR degradation rate. However, there is an interesting phenomenon: When we knocked down TPO or overexpressed TPO in EGF‐stimulated NSCLC cells, the EGFR level did not change notably, whereas the P‐EGFR level was altered significantly. This suggests that a negative feedback mechanism exists as EGFR mRNA level changes in a manner opposite to that of TPO and P‐EGFR levels. When the EGFR degradation rate is changed continuously, NSCLC cells tend to compensate by modifying the EGFR transcription process.

EGFR‐targeted therapy has become a first‐line treatment as it considerably improves the clinical outcome of NSCLC patients with sensitizing EGFR mutations. Mutant EGFR is constitutively activated and initiates ligand‐independent signalling, resulting in high EGFR signalling and enhanced endocytic trafficking in NSCLC cells.^33^ As our results showed that TPO promotes NSCLC progression by regulating EGFR degradation, we hypothesize that TPO could be a possible therapeutic target for treating NSCLC. In particular, the combination of EGFR‐targeted and TPO‐targeted therapy may have greater clinical efficacy. TPO‐targeted therapy is not a new concept; besides haematopoietic system diseases, it has been studied and developed to assist in treating some solid tumours such as ovarian tumour and breast tumour.^34^, ^35^ However, these current studies targeted the exogenous TPO produced in the liver or kidneys, aiming to counter the paraneoplastic thrombocytosis, which may lead to platelet‐dependent cancer progression. For NSCLC treatment, it will be quite different as anti‐TPO treatment is supposed to specifically target the endogenous TPO in NSCLC cells.

Our study encountered some limitations. We were unable to analyse the prognostic information for the specimens used for immunohistochemistry as these samples were collected from 2014 to 2016 and we had insufficient time to gather this information. EGF stimulation was performed as agonist of EGFR signalling and to amplify ligand‐induced EGFR activation and endocytic trafficking; however, the mechanisms induced by low or saturated EGF concentrations are not consistent. Two different entry routes are involved in the internalization step: (a) Clathrin‐mediated endocytosis (CME) is activated in all cell types under all ligand concentrations and mainly tends to recycle EGFR; (b) non‐clathrin‐mediated endocytosis (NCE) is activated under high ligand concentrations in specific cellular conditions and targets the majority of activated EGFR to the lysosome for degradation.^36^, ^37^, ^38^ These two internalization pathways always occur simultaneously at a certain ratio, which determines the final signalling response; thus, under different ligand concentrations, the ratio of these two pathways differs. However, we were unable to take this uncertainty into account during our study. Determining the precise mechanism and exact timing of every step of EGFR endocytic trafficking has always been the main challenge in EGFR signalling research.

## CONFLICT OF INTEREST

The authors confirm that there are no conflicts of interest.

## AUTHORS' CONTRIBUTIONS

XS conceived the study and supervised the experiments; ZZF conducted the experiments and wrote the manuscript; FXX and LY contributed to clinical specimen collection and performed immunohistochemistry; SYB and SGH contributed to cell culture, proliferation assay and migration assay; and ZX and LXH contributed to RT‐PCR and statistical analysis. All authors read and approved the final manuscript.

## Supporting information

Fig S1

Fig S2

## Data Availability

The data used to support the findings of this study are available from the corresponding author upon request.
