# Supplementary material for: Endogenous thrombopoietin promotes non‐small‐cell lung carcinoma cell proliferation and migration by regulating EGFR signalling
Source: J Cell Mol Med. 2020 Apr 26;24(12):6644–57. doi: 10.1111/jcmm.15314 (PMC7299695; doi:10.1111/jcmm.15314)
Supplement: Supplementary file 1 — Fig S1 [file JCMM-24-6644-s001.docx]

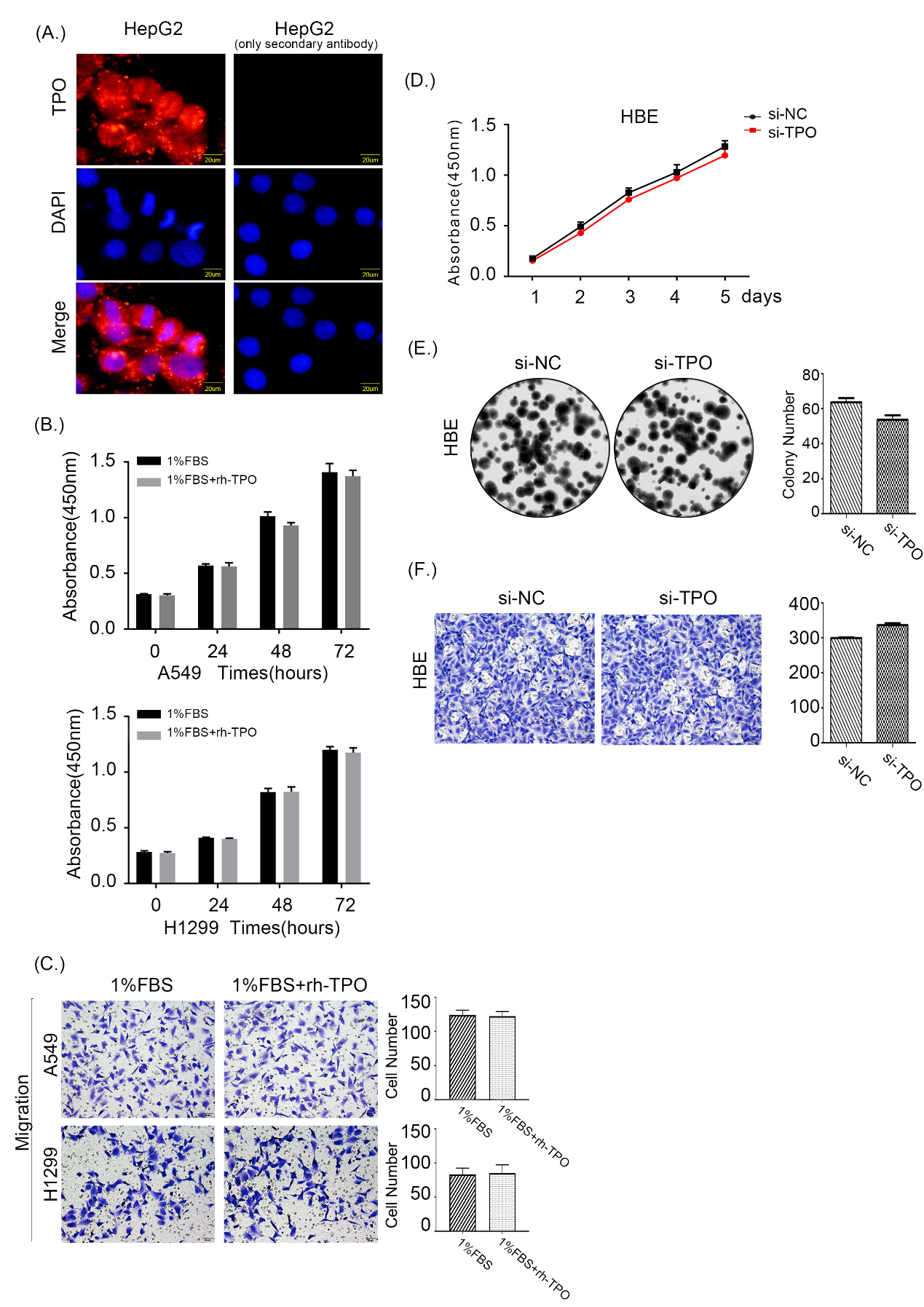


**Supplementary Fig. S1.** **A**, Immunofluorescence assays demonstrated the expression of TPO in HepG2 cells as the positive control. Only secondary antibody without TPO antibody served as a negative control. Magnification, ×400. **B**, A549 and H1299 cells were stimulated with rh-TPO (50 ng/ml) for 24, 48, and 72 h. CCK8 assays showed that there was no influence on the proliferation of these two cells. **C**, A549 and H1299 cells were stimulated with rh-TPO (50 ng/ml) for 24h. Transwell assays showed that there was no influence on the migration of these two cells. **D, E**, CCK-8 and colony formation assays demonstrated that suppressing TPO does not affect the proliferation of HBE cells. **F**, Transwell assays demonstrated that suppressing TPO does not affect the migration of HBE cells. Data are presented as the mean ± SD of three independent experiments.
