# Supplementary material for: Endogenous thrombopoietin promotes non‐small‐cell lung carcinoma cell proliferation and migration by regulating EGFR signalling
Source: J Cell Mol Med. 2020 Apr 26;24(12):6644–57. doi: 10.1111/jcmm.15314 (PMC7299695; doi:10.1111/jcmm.15314)
Supplement: Supplementary file 2 — Fig S2 [file JCMM-24-6644-s002.docx]

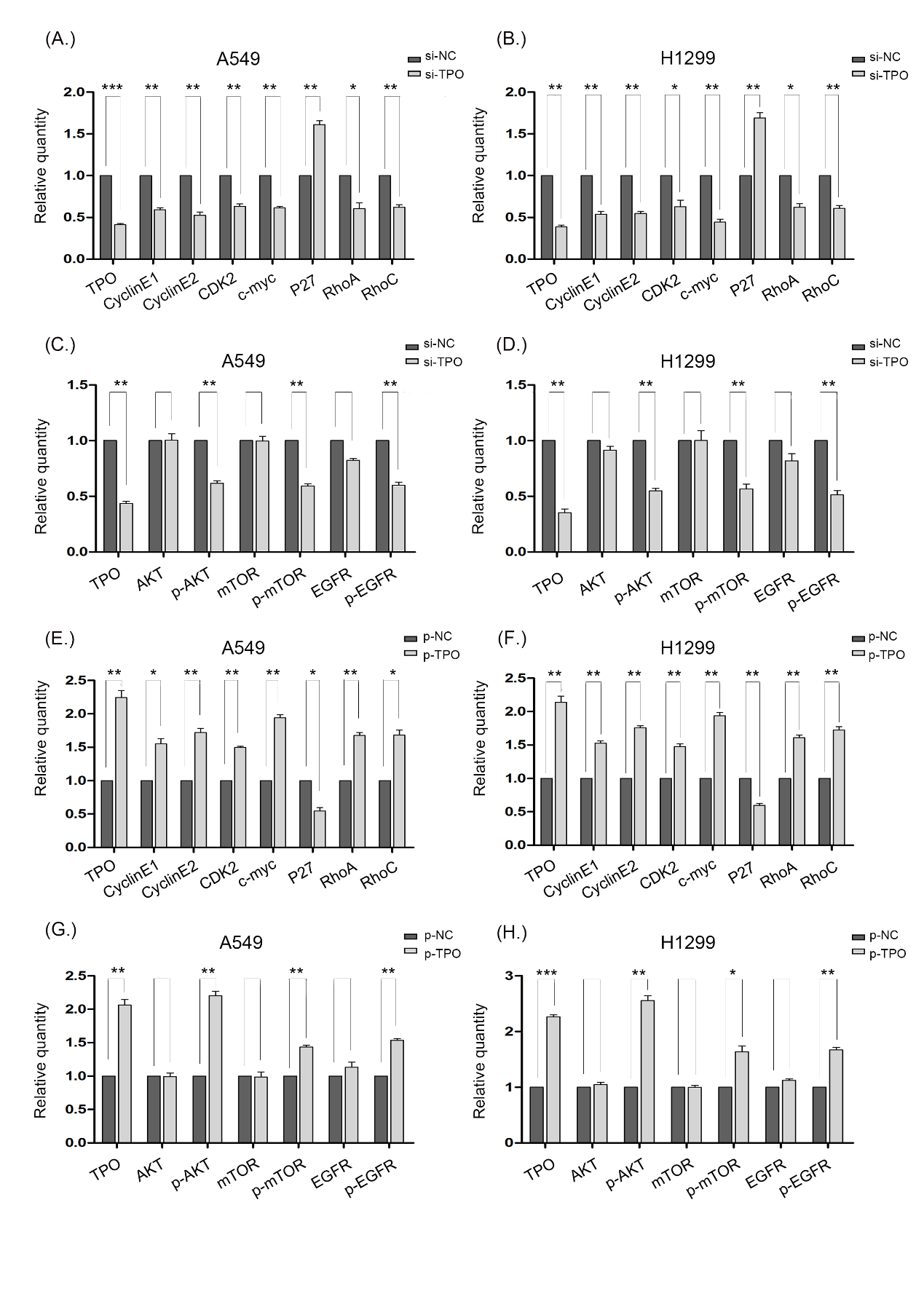


**Supplementary Fig. S2.** **A-D**, Relative quantification of western blot bands in Figure 3. **E-H,** Relative quantification of western blot bands in Figure 6. All the relative quantification of western blot bands is based on grayscale values analyzed by Image J software. The value of the control group was set to “1”. **P* < 0.05; ***P* < 0.01; ****P*< 0.001. Data are presented as the mean ± SD of three independent experiments.
